# First dorsal compartment musculotendinous avulsion accompanied by close radial styloid fracture: Case report

**DOI:** 10.1016/j.ijscr.2018.10.007

**Published:** 2018-10-24

**Authors:** Emre Anıl Özbek, Mehmet Armangil, Sırrı Sinan Bilgin

**Affiliations:** a Yozgat City Hospital, Orthopedics and Traumatology Department, Yozgat City Hospital, 66100 Viyana Avenue, Yozgat, Turkey; b İbn’i Sina Training and Research Hospital, University of Ankara, Orthopedics and Traumatology Department, İbn’i Sina Hospital, Ankara University Medicine Faculty, 06100, Samanpazarı, Ankara, Turkey

**Keywords:** APL, EPL, First dorsal compartment, Musculotendinous avulsion, Radial styloid fracture

## Abstract

**Introduction:**

Radial styloid fracture and concomitant first dorsal compartment proximal musculotendinous avulsion is extremely rare injury. This togetherness is difficult to diagnose fully on routine physical examination.

**Presentation of case:**

In this study, we present a thirty nine year old male patient who suffered musculotendinous avulsion injury of the extensor pollicis brevis (EPB) tendon and abduc- tor pollicis longus (APL) tendon that is rarely accompanied by a closed, non-displaced radial styloid fracture developed following an in-car traffic accident. Diagnosis of avulsion was made with preop- erative magnetic resonance imaging (MRI) and the fracture was fixed with open reduction. The measurements of isometric APL and EPL muscle strength for two thumb were performed using a digital hand dynamometer, no statistically significant difference was found between the muscle strengths of the affected and non-affected thumbs at the postoperative second year follow- up (p > 0.05).

**Discussion:**

The literature does not hold enough cases to establish the grounds for hypotheses related to the injury mechanism in the 1 st extensor tendon musculotendinous injuries ac- companying radial styloid fractures. Although the diagnosis of the injury in our case was inadver- tently made with preoperative MRI, the routine application of MRI does not seem to be cost-effec- tive.

**Conclusion:**

We suggest that checking and assuring the intactness of the 1 st extensor compartment with a gentle traction during surgery should be a routine step in the treatment of radial styloid fractures treated with open reduction.

## Highlights

• Our study is the third case report about radial styloid fracture accompanying with first extensor compartment muscles rupture.
• Our study will be the first study in this issue because of some privileges.
• Other case report did not have long term follow-up time, mean that just 8 month follow-up time.
• Our study also has preoperative MRI views and intraoperative photographs.
• First case report; which was about this issue, muscle strength test was not applied by authors.

## 1 Introduction

Distal radius fractures are the most common long bone fractures [1] and the most common tendon injury accompanying these fractures is the injury of the extensor pollicis longus (EPL) tendon [2]. However, distal radius fractures concomitant with avulsion injuries of the EPB and APL tendons of the 1 st dorsal compartment have been rarely reported in the literature [2].

In our case report, the results of the muscle strength analysis and two years of follow-up of a patient with a non-displaced fracture of the radial styloid and musculotendinous avulsion injury of the 1st dorsal compartment tendons are presented. SCARE checklist was performed for this study [3].

## 2 Case report

**Fig. 1:**
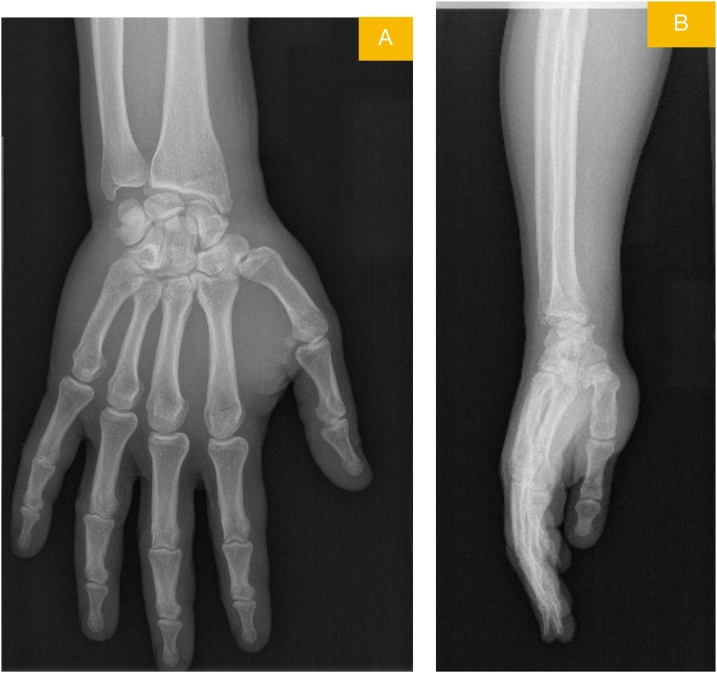
Posteroanterior (A) and lateral (B) view radiographs of wrist showing radial styloid. displaced and ulnar styloid minimally displaced fractures

**Fig. 2:**
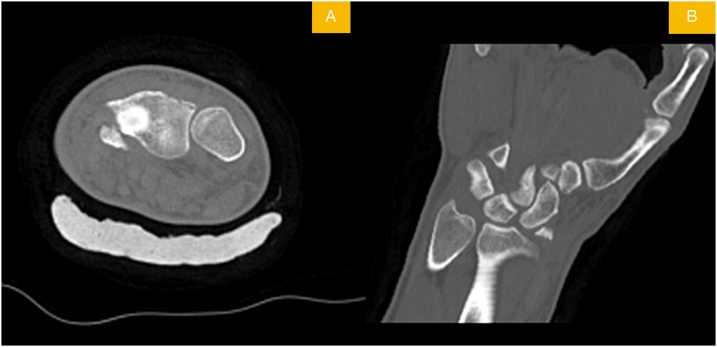
Axial (A) and coronal (B) CT scan views of displaced radial styloid fracture.

A 39-year-old male patient presented to the emergency room due to pain and swelling in the dominant right wrist, following an in-car traffic accident. His physical examination revealed limited and painful movement in the right wrist and tenderness over the radial and ulnar styloids. No ne- urovascular deficits were detected. The patient did not have a history of any disease. A non-displa- ced radial styloid fracture and a minimally displaced fracture of the ulnar styloid were observed on the anteroposterior and lateral X-ray view of the wrist (Fig. 1). The patient underwent MRI in the emergency room, as a requisite of a M.D. thesis planned to be published later (Fig. 2). The radi- ology department of our hospital reported findings of “rupture and retraction in the APL tendon” in the MRI report. Based on these findings, the previously intended surgical treatment of the non-disp- laced fracture with closed fixation was switched to open reduction and fixation.

**Fig. 3:**
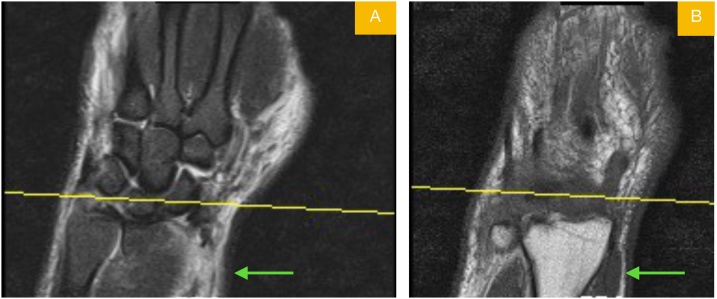
First dorsal compartment retraction coronal view of MRI T1 (A) and T2 (B) sequences.

**Fig. 4:**
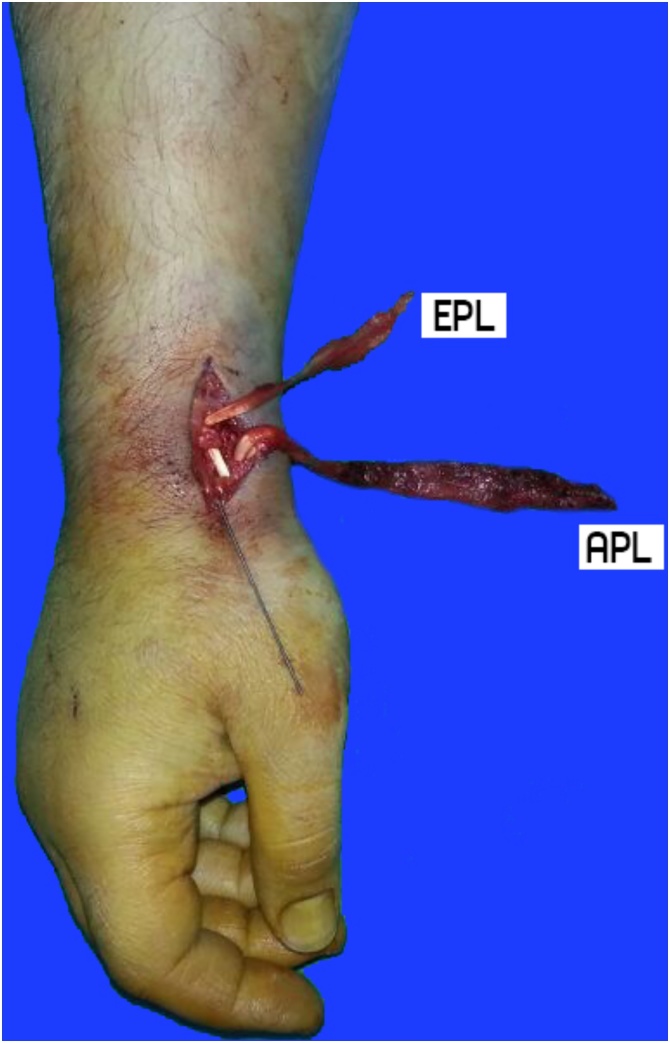
Intraoperative photo of extansor pollicis brevis(EPB) and abductor policies longus(APL). musculotendinous injuries

Following general anesthesia, the patient was operated via the longitudinal dorsal approach over the anatomical snuff box. A single EPB and two APL tendons were observed in the 1 st dorsal compartment, as reported in 70% of the cases in the literature [4]. Following gentle traction with a tendon hook, the musculotendinous avulsion was visualized on the proximal aspect of the tendons (Fig. 3). The radial styloid fracture of the patient was fixed using a cannulated compression sc- rew (Medartis®; Germany) (Fig. 4). The tendons were cut off the musculotendinous juncture and the tendon stumps were left free beneath the forearm fascia for a probable tendon transfer in the fu- ture. The patient was postoperatively followed with a palm-based thumb spica splint for three week and then hand and wrist exercises were performed to the extent the patient could tolerate. The pati- ent exhibited no limitation of movement or functional loss throughout the two-year follow-up peri-od.

**Fig. 5:**
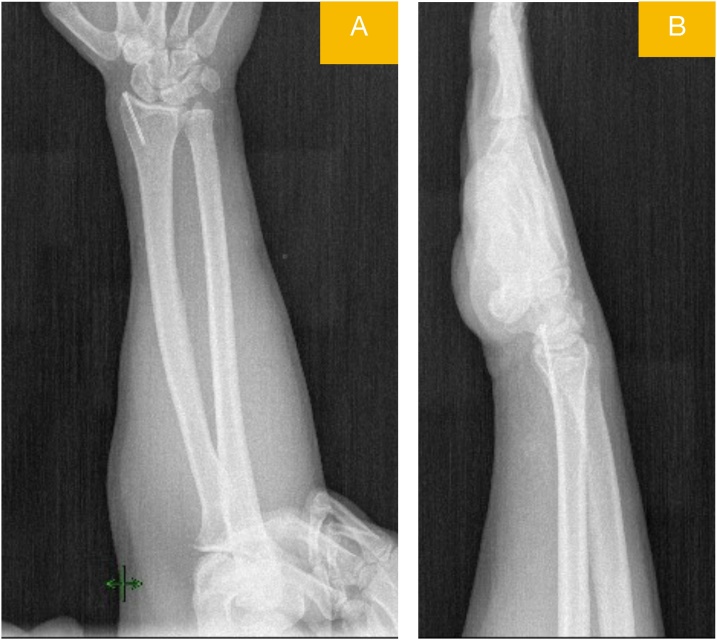
Posteroanterior (A) and lateral (B) views of postoperative second year wrist radiographs.

The abductor and extensor muscle strengths of the operated and non-operated thumbs were objectively measured at the second year follow-up. Ethical approval had not been applied by authors for this study. The measurements of isometric muscle strength were performed using a digital hand dynamometer MicroFet 2 (Hoggan Health Industries, Draper, UT), used safely in numerous studies, and the muscle strengths were recorded in Newtons (N) (Fig. 5) [[5], [6], [7]]. Patients were verbally encouraged and asked to exert full force on the dynamometer for 5–10 seconds. The process was repeated five times with two-minute intervals of rest[5,6,7].

The paired sample *t*-test was used in comparing the extensor and abductor muscle strengths of both thumbs. The mean extensor force exerted by the affected thumb was 13.48+-0.36 N and 13.44+-0.36 N for the left thumb. The mean abductor force exerted by the affected thumb was 9.3+-

2.23 N and 12.22+-0.9 N for the left thumb. No statistically significant difference was found in the extensor and abductor muscle strengths between the two thumbs (p > 0.05).

## 3 Discussion

**Fig. 6:**
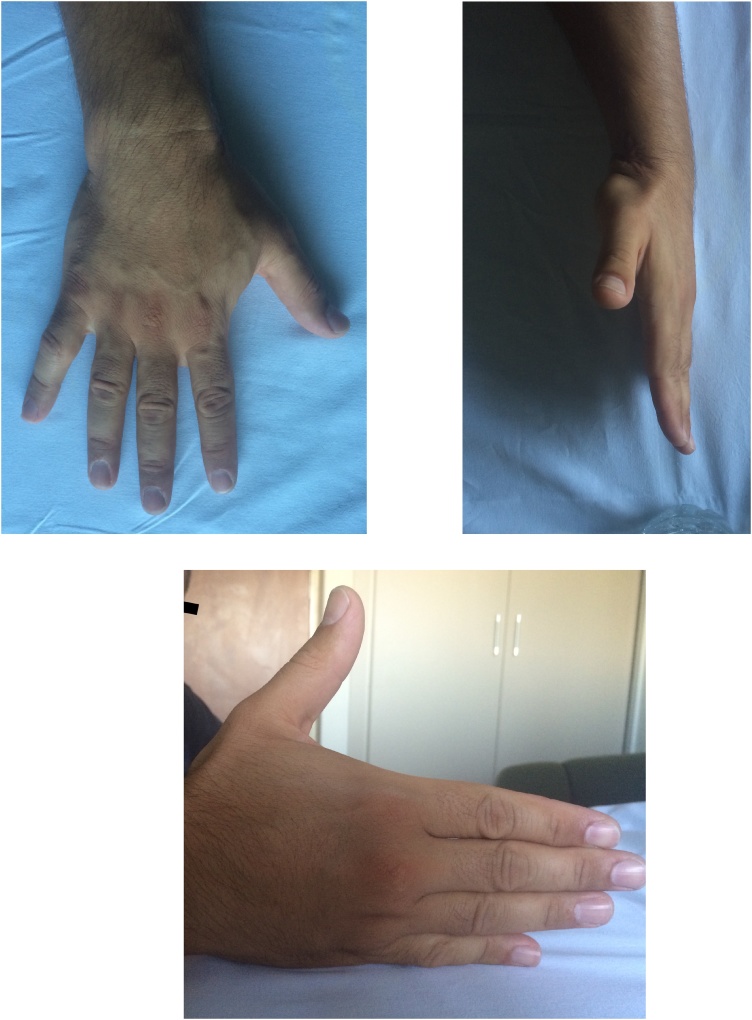
Clinical photos of last control (2-year follow up).

**Fig. 7:**
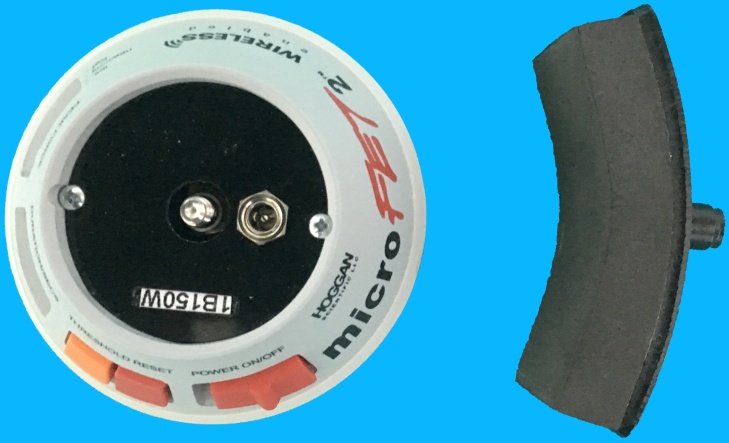
Digital hand-held dynamometer MicroFet 2 (Hoggan Health Industries, Draper, UT).

The literature holds only one study reporting a radial styloid fracture concomitant with mus- culotendinous avulsion injury of the 1 st dorsal compartment tendons of the wrist [2]. On the other hand, studies that report no functional loss in the long-term follow-up of 1 st dorsal compartment in- juries which require no tendon transfer or other type of reconstruction also exist [2,8]. Our report presents a rarely seen case in the literature and can be deemed unique considering the presence of preoperative MR images and the postoperative objective measurements of the muscle strengths (Fig. 6, Fig. 7).

In DiMauro et al.’s study planned in parallel to ours, the authors reported a decreased range of motion (ROM) of the joints and a decrease in muscle strength in the long term following muscu- lotendinous avulsion injuries [2]. The authors also hypothesized that the tendon stump scars could lead to chronic complications such as tendinosis. Our patient was followed up for a period of two years and objective muscle measurements showed no loss of strength or the presence of tendinosis or any other chronic complication during this period.

Upon a review of the literature, we found one case report investigating the loss of function in thenar motor units in patients whose tendons of the 1 st extensor compartment were excised due to open musculotendinous avulsion of the APL and EPB tendons [8]. The authors compared hand grip, thump ROM and pinch strengths and found no difference between the two hands in terms of st- rength. However, a decrease was detected in the radial extension ROM of the 1 st digit in the injured hand but was considered insignificant as it was expected to cause no loss of functional in the pre- sence of other wrist stabilizers and muscles. In parallel to this study, our patient exhibited no loss of functional movement in the long-term follow-up.

In our case, the two APL tendons were observed intraoperatively. The condition is reported to have a prevalence of 70% in general population and mostly manifests itself bilaterally [4]. In addition, there is a high risk of injury to the supernumerary tendons and De Quervain’s stenosing te- novaginitis during surgical approach [9]. In our study, however, no tendon injury during surgical approach nor De Quervain’s stenosing tenovaginitis in the contralateral wrist was observed in our patient in the long-term follow-up.

The literature does not hold enough cases to establish the grounds for hypotheses related to the injury mechanism in the 1 st extensor tendon musculotendinous injuries accompanying radial sty- loid fractures. Although the diagnosis of the injury in our case was inadvertently made with preope- rative MRI, the routine application of MRI does not seem to be cost-effective. However, following our experience with the above case, checking and assuring the intactness of the 1 st extensor com- partment tendons with gentle traction has been added as a routine step to our surgical procedures for all patients with radial styloid fractures treated with open reduction.

## Patient consent

Written informed consent was obtained from the patient for publication of this case report and accompanying images. A copy of written content is available for review by Editor-in*Chief of this journal on request.

## Provenance and peer review

Not commissioned, externally peer reviewed.

## Conflicts of interest

Any authors has not any disclosure.

## Sources of funding

Any fundings were not needed for our study

## Ethical approval

Ethical approval has been exempted by our institution.

## Consent

The fully informed written consent were signed by case patient of our study.

## Author contribution

Emre Anıl ÖZBEK, M.D. : Corresponding author, data collection, data analysis, writing the paper Mehmet ARMANGİL, M.D.: Writing the paper, study design

Sırrı Sinan BİLGİN, M.D.: Study design

## Guarantor

Emre Anıl ÖZBEK, M.D.
